# The effect of different combinations of physical activity and natural environment videos on children's attention levels between class breaks

**DOI:** 10.1186/s12887-023-03868-8

**Published:** 2023-02-03

**Authors:** Xiao Luo, Meng Tao, Jiahao Lu, Li Lu, Xiaolong He

**Affiliations:** 1 School of physical education and health science, Zhejiang Normal University, Jinhua, China; 2 Zhejiang Guangsha Vocational and Technical University of Construction, Jinhua, China; 3 Taizhou Vocational College of Science & Technology, Taizhou, China

**Keywords:** Exercise, Virtual reality, Children, Attention

## Abstract

**Background:**

Physical activity intervention and watching natural environment videos have been proven to improve young children’s attention levels. However, evidence comparing the improvement effects of different combinations of the two activities has rarely been reported. By comparing the differences in the improvement effects of four combinations of physical activities and watching natural environment videos on young children’s attention levels, this study can enrich the evidence in this research field and also provide a reference for arranging effective intervention methods for children’s attention recovery between classes.

**Method:**

A total of 152 children aged 4 to 6 years were recruited and randomly divided into four intervention groups: (1) physical activity intervention first and thereafter watching a natural environment video group (activity + video group), (2) watching a natural environment video first and thereafter the physical activity intervention group (video + activity group), (3) physical activity-based group, and (4) natural environment video-based group. Physical activity involved 4 min of moderate-intensity basic physical fitness combination training. The subjects wore the Pico Neo pioneer version of the VR glasses all-in-one machine to watch a natural environment video. Thereafter, population sociological variables and daily physical activity levels were investigated. Auditory and visual sustained attention tests were performed before and after intervention in each group.

**Result:**

The auditory attention post-test scores of the four groups showed an improvement trend compared with the pretest scores. In particular, the activity + video group (*F *= 10.828; ɳp2 = 0.226; *p* = 0.002) and natural environment video-based group (*F* = 9.452; ɳp2 = 0.203; *p* = 0.004) have the best improvement effect. For visual attention, only the activity + video group showed a significant improvement trend (*F* = 4.287; ɳp2 = 0.104; *p* = 0.045), while the other three groups showed a downward trend in scores.

**Conclusions:**

Among the different intervention combinations, the physical activity intervention first and watching natural environment videos thereafter group has the best effect on improving children’s attention during recess. Physical activity interventions at the end of recess adversely affect young children’s visual attention levels at the beginning of the class. Therefore, this study recommends that children should not engage in physical activity interventions in the second half of the class break. Lastly, the current research recommends presenting the content of physical activity interventions first and further improving their attention thereafter by watching natural environment videos.

## Background

To better interact with stimuli in the environment, people often selectively process information most relevant to tasks; this mechanism is often referred to as “attention” [1]. Attention is the ability of individuals’ mental activities to be directed and focused on something, and studies have shown that good attention helps people learn and work efficiently [2]. In general, attention can be divided into selective and sustained attention. Selective attention refers to the ability to select relevant stimuli from the environment, whereas sustained attention refers to the ability to remain focused over a certain period [3]. However, people’s attention levels at work and study tend to decrease with increasing fatigue, possibly hindering individual work and study efficiency [4]. Therefore, exploring ways to improve attention between work and study has positive implications for enhancing people’s efficiency at work and study [5].

Related empirical studies have shown that physical activity interventions help improve individual attention levels [6]. From the molecular biology perspective, a certain intensity of physical activity has been found to help the release of insulin growth and brain-derived neurotrophic factors, which can promote the expression of related genes, thereby achieving the effect of improving concentration [7, 8].Wood et al. (2020) showed that physical activity has a significant positive correlation with young children’s cognitive functions, particularly self-regulation, sustained attention, and working memory. Moreover, several short intervention studies have confirmed the positive effects of physical activity interventions on promoting attention improvement [9, 10, 11]. Attention recovery theory divides attention into intentional and unintentional attention, and the attention people use in natural environments, such as watching trees, blue skies, and lakes in natural environments, is dominated by unintentional attention [12]. People’s contact with the natural environment, such as viewing pictures of the natural environment and entering green spaces for relaxation activities, will increase unintentional attention and reduce attention fatigue caused by prolonged intentional attention [13].

With the rapid development of technology, virtual reality (VR) technology provides a new and effective way for the public to get in touch with green space [14]. VR technology is a medium consisting of interactive computer simulations that give a sense of mental immersion in the simulation [15]. Moreover, the virtual natural environment can achieve a useful complement to the real natural environment [16]. As research continues, combined visual and auditory interventions that incorporate physical activity and viewing videos of the natural environment can help further improve attention levels [17, 18, 19]. In addition to providing the public with new and effective ways to view green videos, VR technology has also shown superior application in the customization of personalized intervention activity programs. Anderson et al. (2017) found that presenting virtual nature scenes using VR technology, when consistent with personal preferences, could help people considerably maintain prolonged concentration, further improving attention [20]. As the hope of future social development, the cognitive development (including attention) of young children in early childhood will have an impact on the level of cognition throughout their lives [21, 22]. At present, VR technology, such as virtual videos of natural environments that can improve attentional resources and cognitive performance, has been shown to be an effective means of enhancing and improving young children’s attention [14, 23].

A survey of kindergarten recess relaxation methods in China found that most kindergartens generally organize outdoor physical activities or watch early childhood educational videos in a uniform manner. Recent studies have analyzed physical activity interventions, exposure to natural environments to improve young children’s attention levels, and the outstanding synergistic effects of the combined interventions [24, 25]. Therefore, this study attempted to use physical activity and green space exposure for attention recovery during class time for young children. However, in the arrangement of kindergarten recess activities, physical activities are often carried out outdoors, while indoor recess is mainly focused on watching educational videos for young children; hence, comparing the way recess content is arranged has a positive effect on improving children’s attention [13, 26]. A search of the literature has revealed insufficient research evidence in this area. The current research referred to previous studies and focused on comparing the effects of four different arrangements of recess relaxation on children’s attention: “physical activity (before) + watching natural environment video (after),” “watching natural environment video (before) + physical activity (after),” “physical activity-based,” and “natural environment video-based.” To ensure consistent experimental environmental conditions, this experiment decided to use VR video indoors as a means of viewing natural environment videos. The experimental results can enrich the theoretical basis of this research area and provide reference and value for the scientific arrangement of kindergarten recess relaxation methods.

## Methods

### Participants

On the bases of the effect sizes and efficacy weights of previous references and experimental research designs [24, 27], this study used a randomized controlled trial grouping and repeated measure analysis of variance (ANOVA) to investigate the differences in the effects of different combinations of physical activities and viewing natural environment videos on improving young children’s attention. To estimate test quantity, this study used G*Power 3.1, the most commonly used test quantity estimation software in the world. In G*Power, the test method was F test, and repeated measure ANOVA, main effects, and interaction effects were selected. Relevant parameters are set as follows: α significance level is 0.05, statistical type is selected as ANOVA: repeated measured, within–between interaction, effect size is selected as 0.25, power weight is 0.80, and the number of measurement groups is 4. Under a normal state of the relevant parameters, output sample size of the power software was 38 people in each group, for a total of 152 people.

**Fig. 1:**
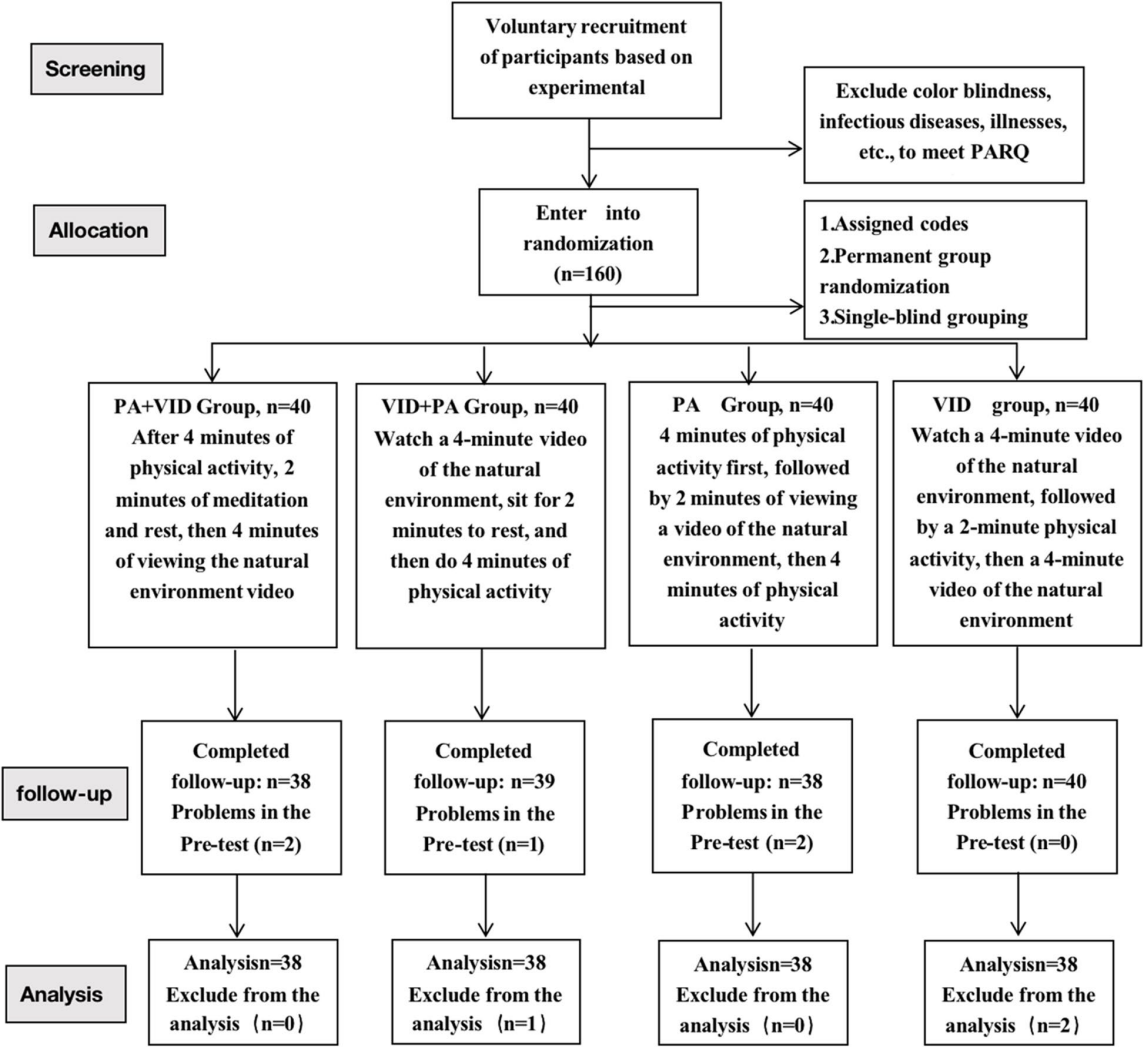
Consort chart showing the recruitment, random allocation, follow-up, and analysis of the participants

The study was conducted in Jinhua City, Zhejiang Province, China. This research was a randomized controlled trial based on several different physical activity and VR natural environment video interventions. Parents and teachers were required to sign an informed consent form before the experiment, and all children were required to obtain their consent to participate. Moreover, the experimental process needed the help of the kindergarten. To further ensure a smooth and successful experimental process, the directors and teachers of the selected kindergartens and our research team had a previous good working relationship. Therefore, our sampling of young children was based on the principle of convenience sampling. Randomized grouping was used in this study, and independent team members who were not involved in other areas of the project were responsible for random assignment. Each recruited participant was assigned a code, and the replacement blocks were randomized to the specified participant’s experimental or control condition after baseline data were pooled. Throughout the study period, team members responsible for randomization were unaware of the participants’ personal data and data collectors were unaware of the participants’ grouping. The recruitment process is shown in Fig. 1. Before the experiment started, we conducted a pretest and found that the attention test used was considerably difficult for children under 4 years old and markedly easy for children over 6 years old, as has been confirmed in previous studies [3]. Hence, we eventually planned to recruit children in the 4–6-year-old age range.

This study was conducted after obtaining ethical approval from Zhejiang Normal University (Project Number ZSRT2022052) before subject recruitment. We communicated with the kindergarten teachers in advance and received their help. The teachers launched the experiment recruitment and informed the experiment protocol in the kindergarten. Through the students’ raising their hands to sign up, informed consent forms were issued to children who volunteered to participate. Thereafter, they were asked to take home the appropriate contents to be filled out by their guardians and the teacher in charge of the respective children. We provided information to the parents and teachers of the subjects on the experiment details.

This experiment eventually recruited 160 small class students in kindergartens in urban areas in Jinhua. To balance the gender factor, we selected the same number of males and females at the time of subject recruitment, and subsequently divided the number of males and females (i.e., 76 each) in each group equally. After excluding 5 subjects who did not complete the experiment and 3 subjects with missing data, 152 subjects completed the experiment and were included in the analysis.

### Physical activity, attention level, and demographics per self-report

To have a complete understanding of the subjects’ individual profiles and their ability to perform daily behavioral activities, demographic and behavioral variables were assessed through a questionnaire, which the teacher in charge of the subject’s classroom was asked to fill out objectively and realistically. The questionnaire includes the following aspects. (A) Personal and family information: age of child, education level of parents, family economic status, and whether or not the child is an only child. (B) Daily behavior survey: daily concentration in class (rating: a. poor; b. generally poor c. better; d. good), number of physical activities during class each week (rating: a. inactive; b. 1 time; c. 2–3 times; d. 4–5 times; e. 6–7 times), number of times viewing videos or pictures of the natural environment each week (rating: a. no viewing; b. 1 time; c. 2–3 times; d. 4–5 times; e. 6–7 times), duration of each viewing (rating: a. 2–5 min; b. 5–10 min; c. 10–15 min; d. 20 min or above), and level of greenery (e.g., lawn, trees, other vegetation) in or around your garden (rating: a. very good; b. good; c. average; d. poor; e. very poor). The experiment could not begin until all questionnaires were completed.

### Experimental process

**Fig. 2:**
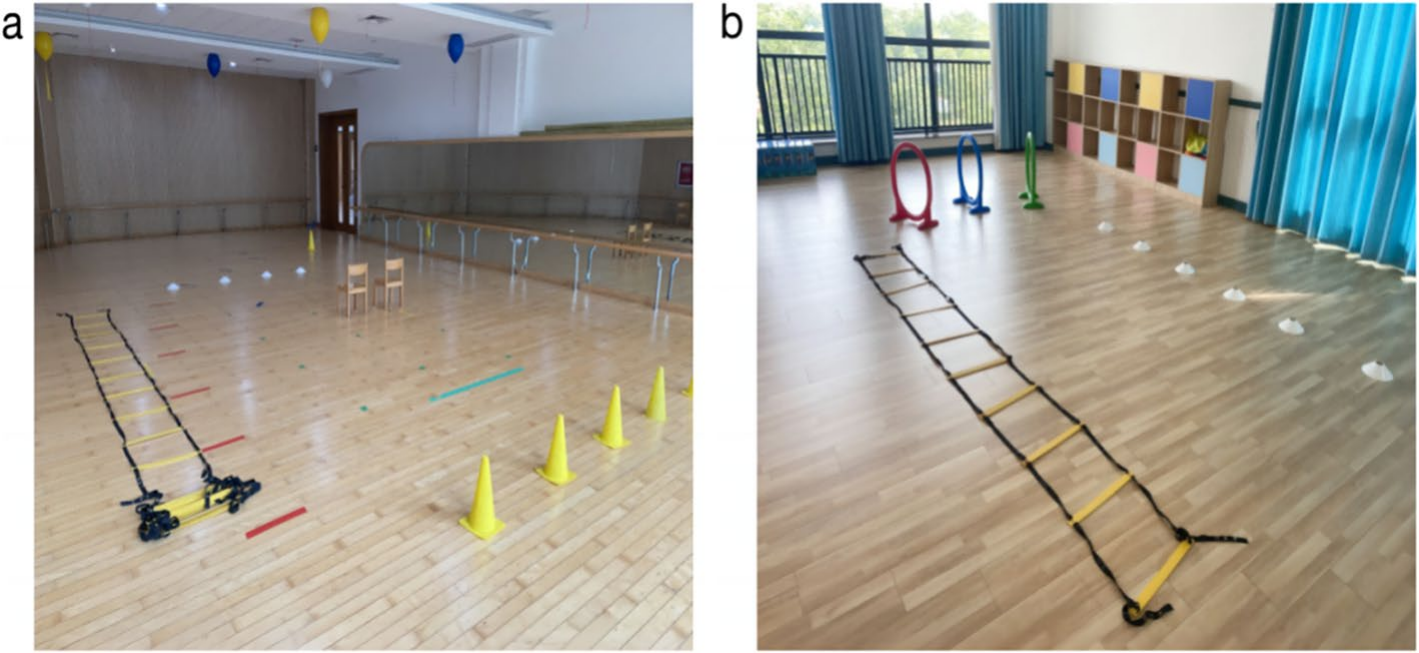
**a** Indoor experimental venue (dance room), **b** activity room

**Fig. 3:**
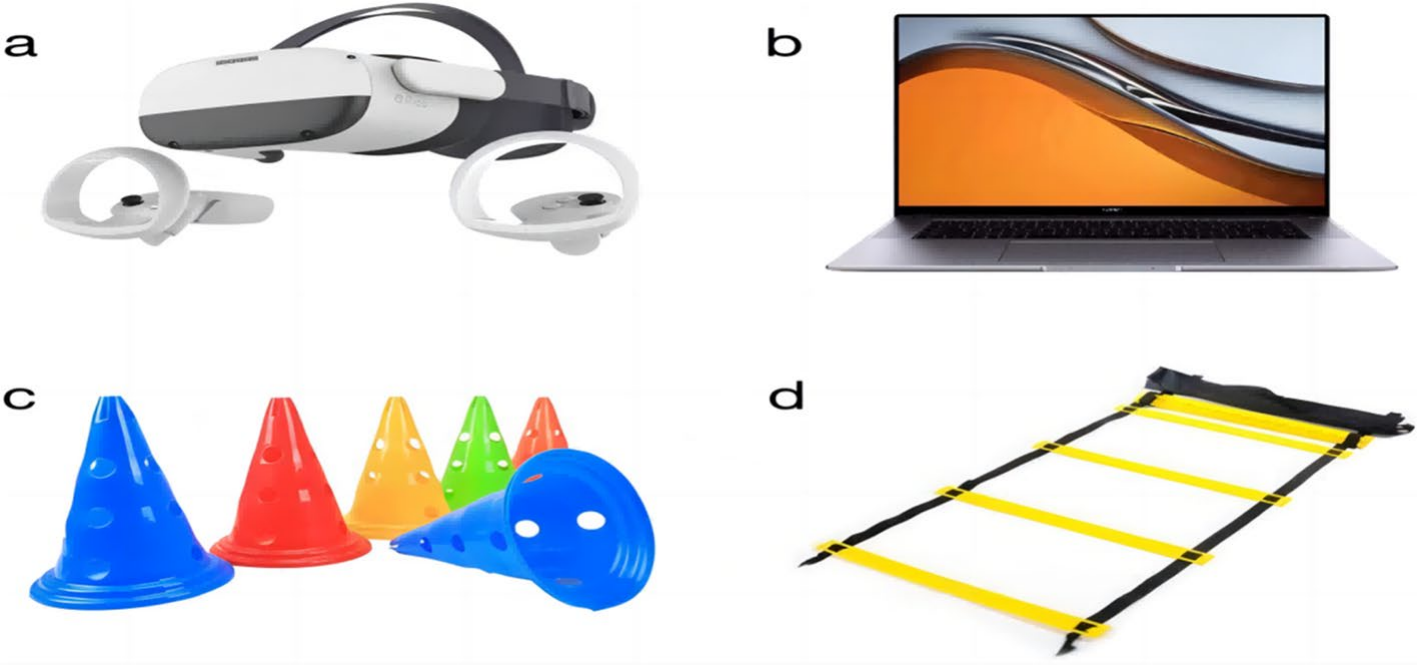
Equipment used in the experiment. The picture was taken from the network. **a** Pico Neo pioneer version VR glasses all-in-one machine, **b** laptop, **c** logo bucket, **d** agile ladder

As shown in Fig. 2, this experimental test was conducted in May and June 2022. The test was conducted in the afternoon after the first class (3–5 pm), and a 10 m × 10 m indoor dance room and a 10 m × 10 m activity room were selected. The test lasted 20–30 min, and there were two sets of experimental equipment and devices (Fig. 3), which were arranged in two rooms far away from each other.

**Fig. 4:**
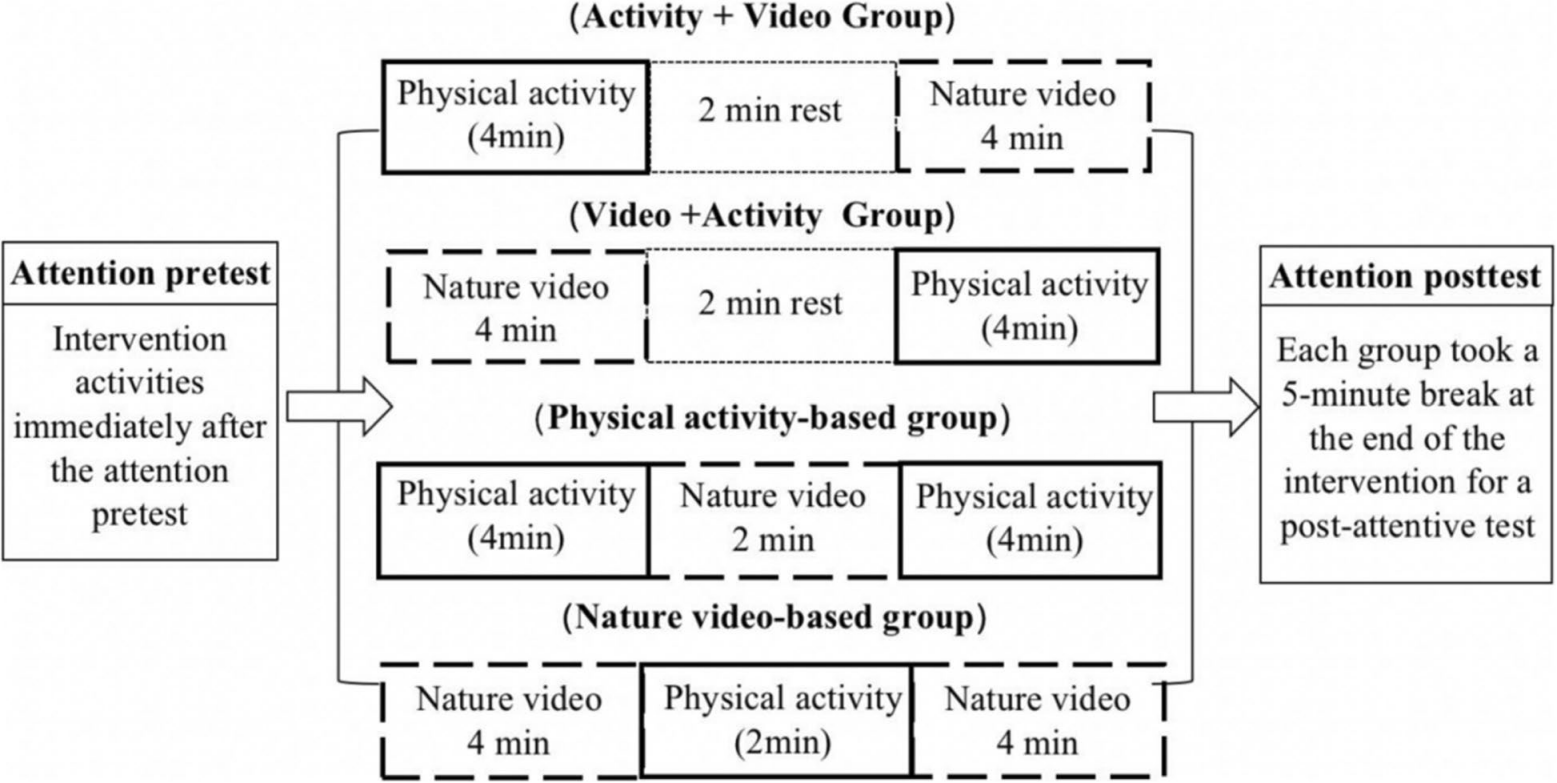
Experimental flowchart

**Fig. 5:**
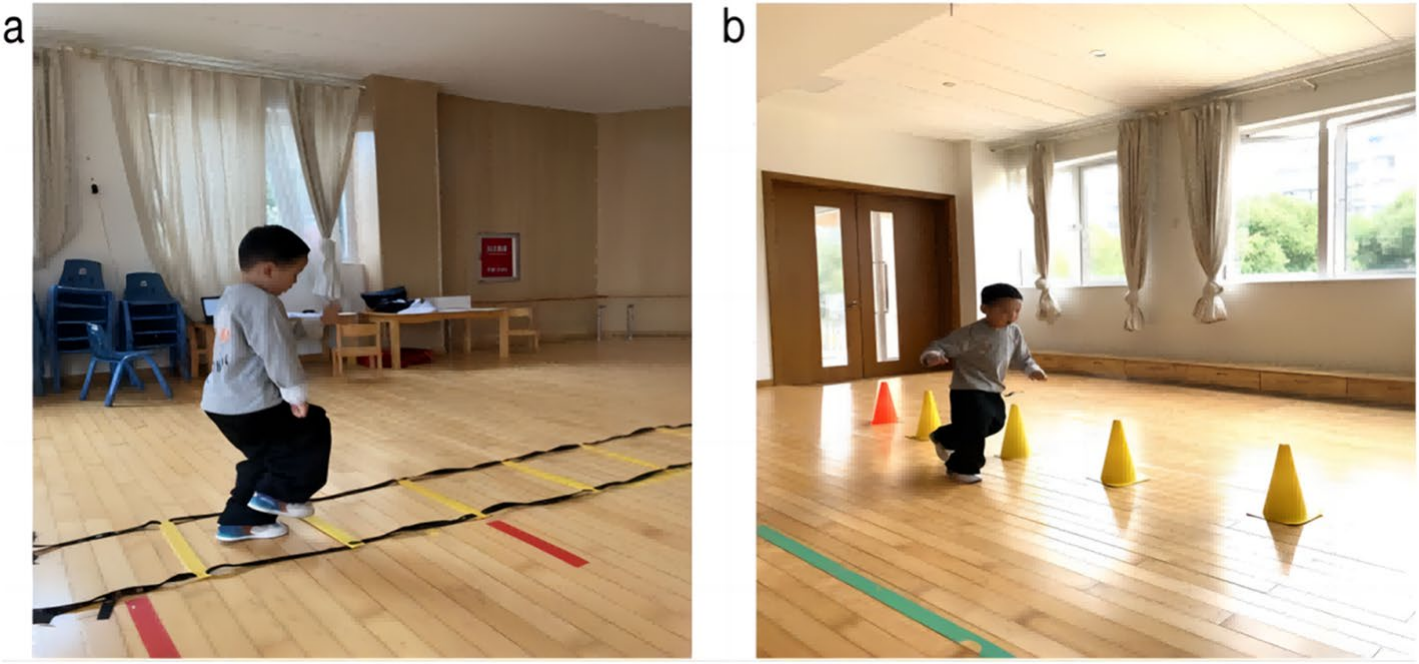
Field photos of physical activity intervention during the experiment

**Fig. 6:**
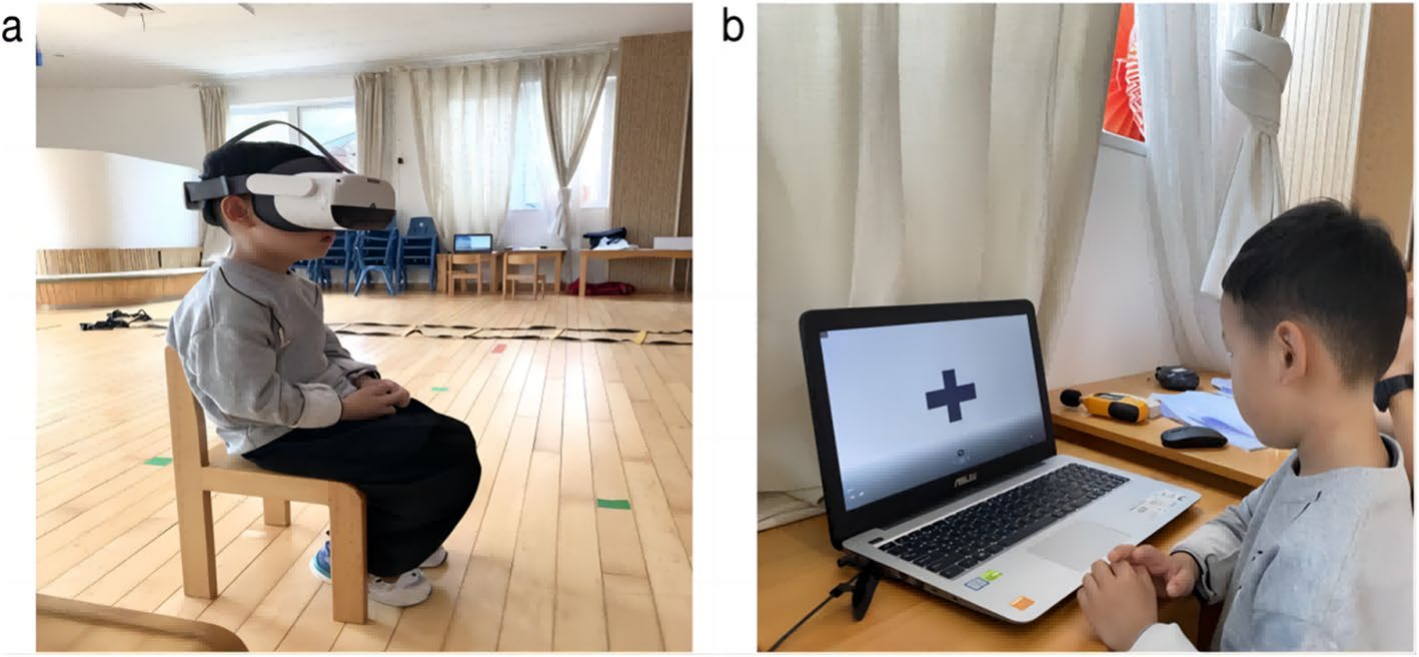
Field photos of the viewing video intervention and attention test during the experiment

**Fig. 7:**
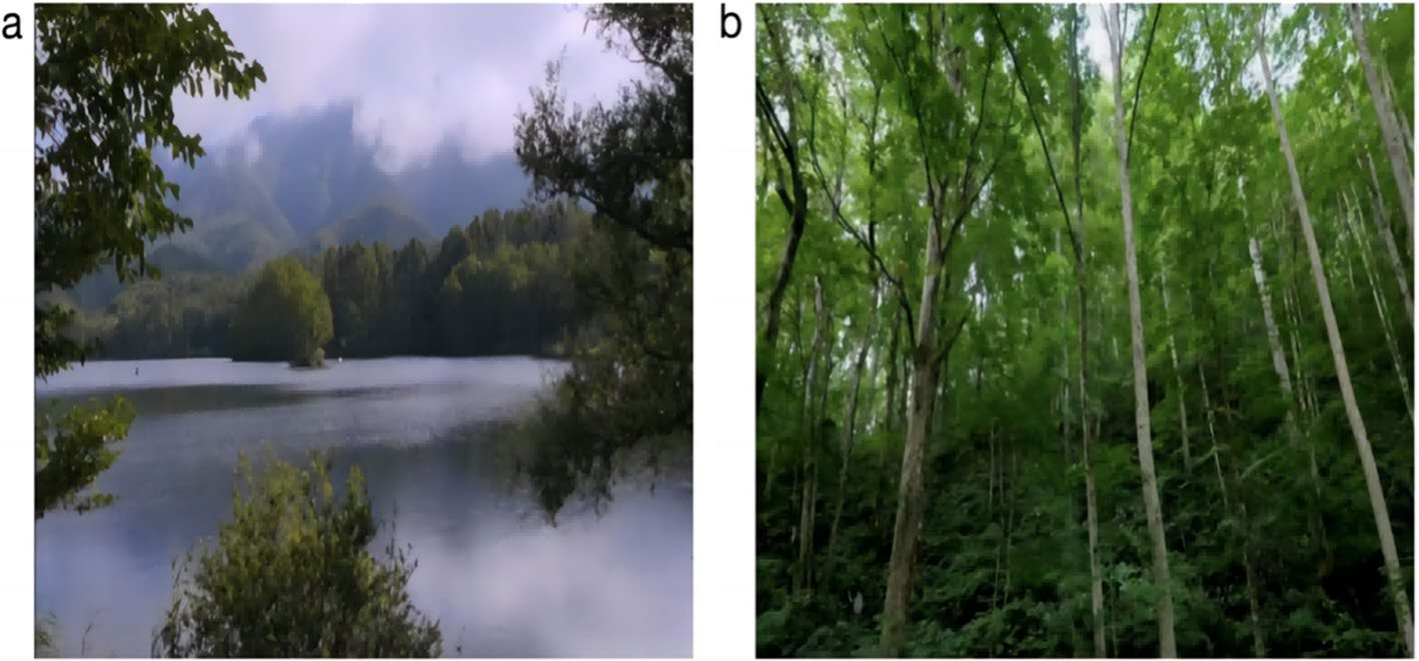
Screenshots of the natural environment video played during the experiment. These pictures were taken from the natural environment video selected by the network

As shown in Fig. 4, this study designed four groups of intervention activities in different combinations, each with a duration of 10 min: simultaneously arranging physical activity intervention and watching natural video intervention, changing the order into an activity + video group and a video + activity group, and arranging a 2-min sitting rest between the two activities. The physical activity group experiment was conducted with a combination of physical activity intervention and watching nature videos as supplement. The nature video group experiment was performed with a combination of watching natural environment videos and physical activity as supplement. (1) Physical activity + nature video group**:** After 4 min of physical activity, sit still for 2 min to rest, and watch the natural environment video thereafter for 4 min. (2) Nature video + physical activity group: Watch a 4-min natural environment video first, sit for 2 min to rest, and perform 4 min of physical activity thereafter. (3) Physical activity-based group: First 4 min of physical activity, then 2 min of nature video, and another 4-min of physical activity, retaining the total length at 10 min. (4) Watching nature videos-based group: Watch a 4-min nature video, followed by 2-min physical activity, and then a 4-min nature video. Given that the subjects of this study were 4–6-year-olds, moderate intensity physical activities that last 4 min and are evaluated with valid intensity indicators are often prone to resistance from parents and kindergartens. We consulted other literature on similar studies of physical activity interventions for young children, which often did not provide validated intensity evaluations of physical activity interventions for young children [28, 29]. The current study conducted a pretest of physical activity prior to the official start of the experiment. By adjusting for this situation, the percentage of heart rate reserve completed by the children in the physical activity content we selected was generally maintained at a moderate intensity. The final design of the 4-min physical activity content was based on basic running, jumping and crawling: jogging in a straight line (5 m) + jogging around a sign barrel (5 m) + jumping on both feet on an agility ladder (5 m) + crawling around a sign barrel (5 m) for 4 min and repeating the cycle. One week prior to the start of the test, the physical activity content was first guided and familiarized by the kindergarten teachers thrice in a uniform manner to ensure a smooth and fluent testing process. After the test started, the research team moderately supervised and guided the subjects to effectively complete the 4-min physical activity. The subjects wore the Pico Neo pioneer version VR glasses all-in-one machine to watch the natural environment video. The video contains green and blue landscapes. The content of each group of viewing videos is the same (see Figs. 5, 6, and 7).

Silence was maintained throughout the entire experimental process because the attention of young children is considerably short, they are easily disturbed by on-site factors, and to maximize the accuracy of the experimental results. Hence, apart from the subjects, the people involved in the experiment and the experimental site staff cannot exceed two people. Before the start of the test, the subjects should be informed of the attention test method and content of the intervention activities in advance. The tester should not communicate or even chat extensively with the subjects, and reduce all behaviors that may distract the subjects’ attention. Attention pre- and post-tests should be carried out for each group of intervention activities. Intervention activities can be carried out immediately after the attention pre-test before the start of each group of intervention activities.

### Test method

This study used the Breckenridge’s (2007) Find Animals task, which is a test of sustained attention and can effectively reflect the ability of children to stay focused over time [3]. The measurement method of this indicator has been applied in numerous studies and its reliability and validity have been confirmed [30]. In the field test, this study will adjust the test appropriately according to the core principles of the animal finding task: shorter stimulus target presentation time and longer total test time are the core principles to increase the difficulty of the test [3].

### Auditory sustained attention test

**Fig. 8:**
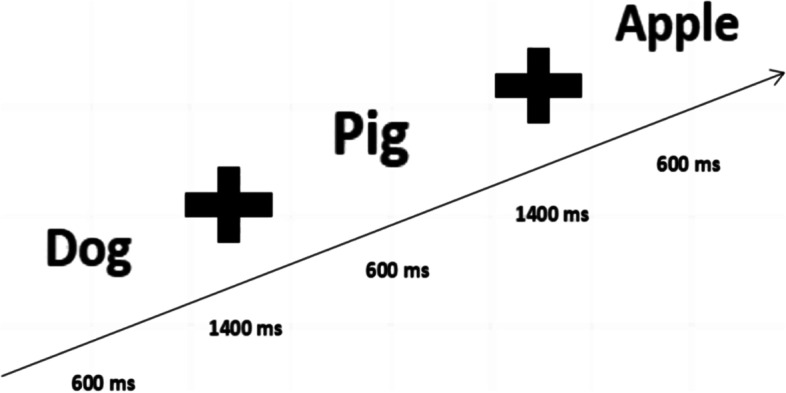
Experimental flowchart of the auditory sustained attention test

**Fig. 9:**
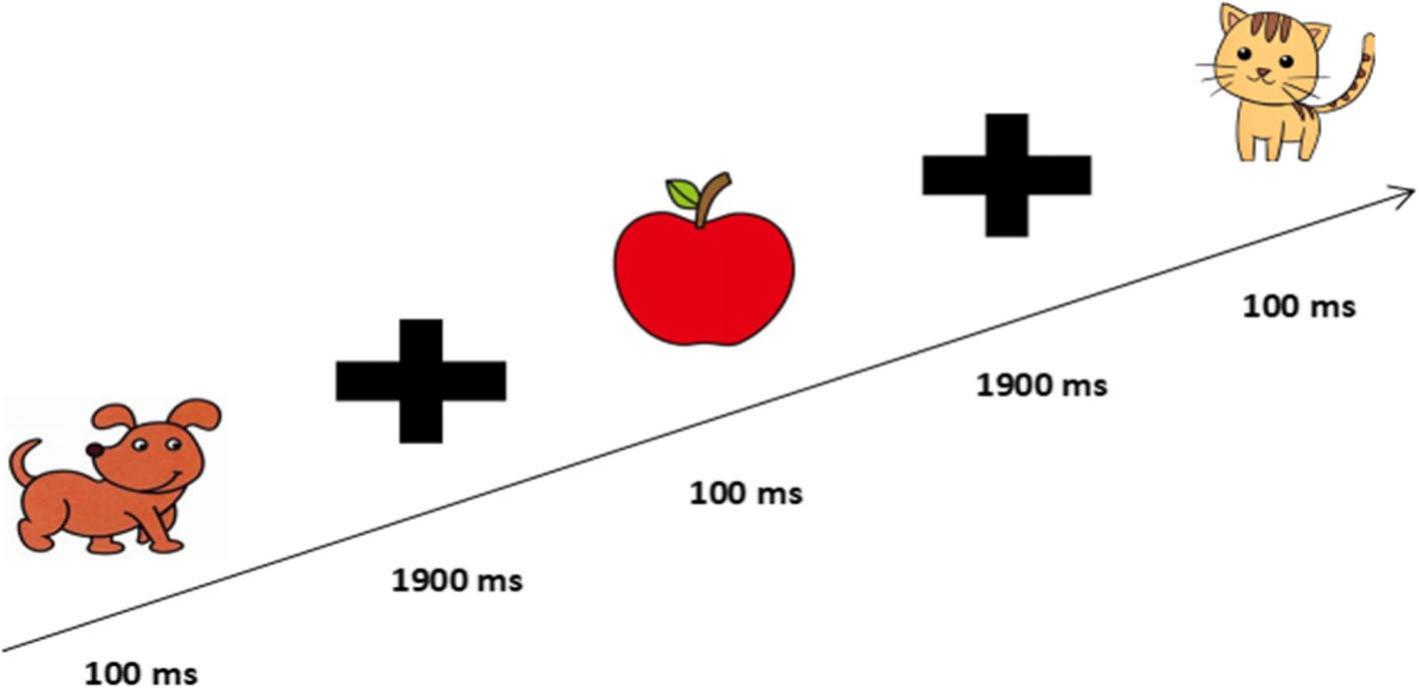
Experimental flowchart of the visual sustained attention test

During the test, the subjects will hear a sample audio that includes the target stimulus (animal name) and non-target stimulus (non-animal name). To reduce the difficulty of naming animals, this study used the most common and single-character animal names (e.g., pigs, cats, dogs, fish, and horses) and non-animal names (e.g., peaches, umbrellas) (Fig. 8). In this study, the stimulus audio presentation time, buffering time (without audio stimuli), and the entire segment time were set to 600 ms, 1400 ms, and 2 s, respectively. The total number of audio presentations for the target stimuli was 20 times, total number of non-target stimuli audio presentations was 80 times, and total number of audio presentations was 100 times, with a total time of 3 min and 19 s (Fig. 9). Before the test, test requirements were explained in detail and a pre-test was conducted. When the target stimulus audio was heard during the test, the animal name should be reported immediately (For children who are more introverted or unwilling to verbally report, they can be asked to make corresponding actions, such as raising their hands and nodding), and hearing non-target stimulus audio was not reported. The tester also immediately reminded the subjects to focus when the target stimulus is missed four consecutive times. The final score for the test is the number of reports to the target stimulus minus the number of reports to the non-target stimulus minus the number of reminders. Once the test started, the subjects were no longer reminded of any behavior unrelated to the test, unless four consecutive correct answers were missed.

### Visual sustained attention test

**Fig. 10:**
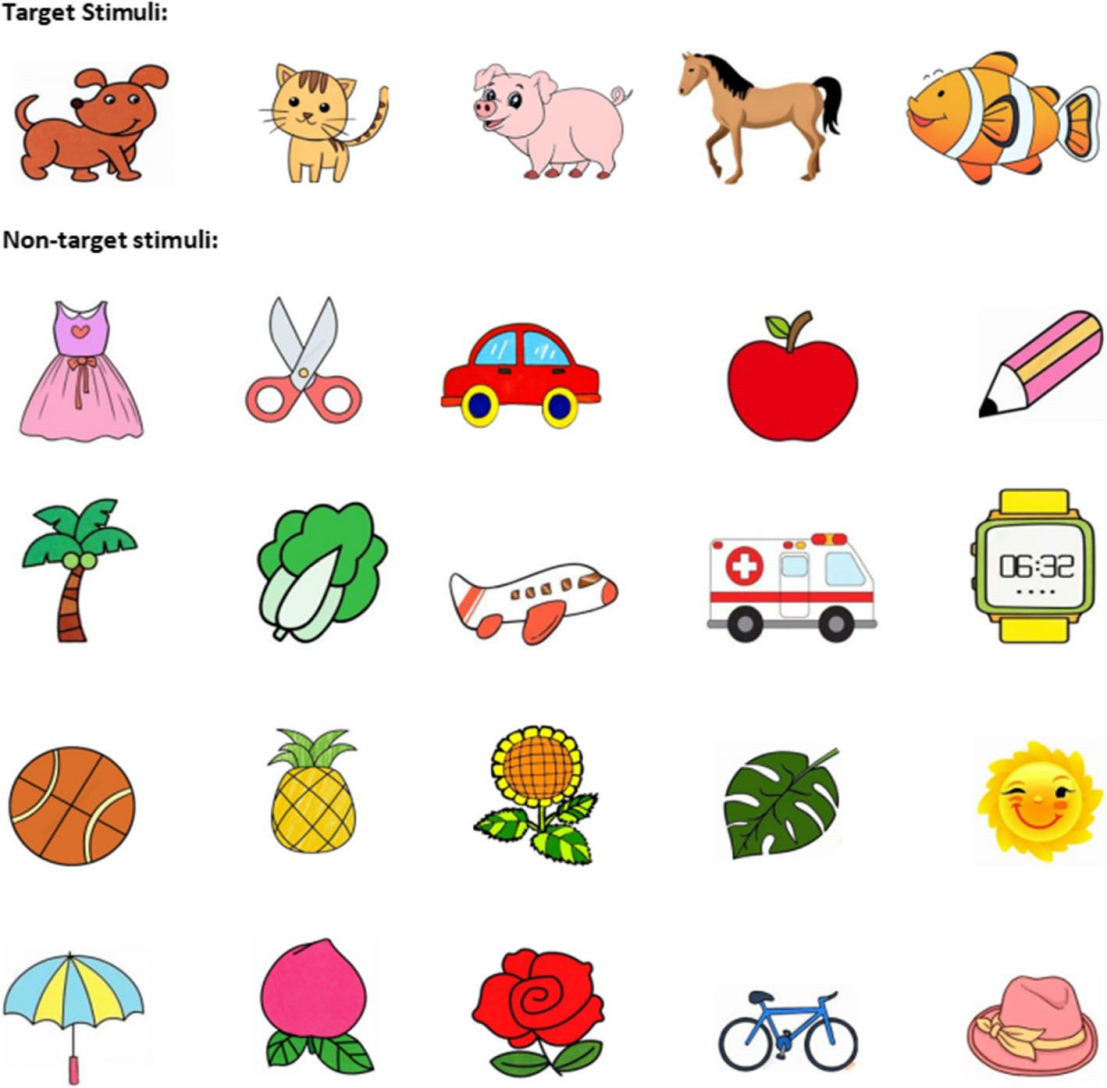
Diagram of the selected items for the attention test

During the test, a series of pictures were presented through a computer or projector, including two categories of target stimulus pictures (animal pictures) and non-target stimulus pictures (non-animal). The content of the pictures presented was consistent with the content of the auditory test (Fig. 8). In this study, the stimulus picture presentation time was set to 100 ms, fixation point (no stimulus) presentation time was 1900 ms, and the entire segment time was 2 s. The stimulus and non-stimulus pictures were presented 30 and 120 times, respectively. The total number of pictures presented was 150 times, and total test time was 5 min (Fig. 10). When the target stimulus picture was seen, the name of the animal should be reported immediately, and the non-target stimulus picture should not be reported. When the report of the target stimulus was missed four consecutive times, the tester immediately reminded the subjects to concentrate. The final score for the test is the number of reports to the target stimulus minus the number of reports to the non-target stimulus minus the number of reminders. During the test, the teacher was responsible for maintaining class order and minimizing uncontrollable factors that interfere with the subjects’ attention level during the process.

### Statistical analysis

At the end of the experiment and completion of data collection, all original data were entered into Excel 2010 for storage. After data entry was completed, the Excel data were imported into IBM SPSS Statistics 21.0 for statistical analysis. Statistical analysis mainly includes the following aspects.

- (1) Descriptive statistical analysis was performed on demographic and sociological variables, such as the ages of children participating in the experiment; and individual factors, such as the level of daily physical activity and frequency of watching natural videos using percentage or mean ± standard deviation.
- (2) Descriptive statistics were used to analyze the pre- and post-measurement values of the dependent variables, such as attention, using mean ± standard deviation. Based on the F-value, p-value, and effect size ɳp2 in the results of multivariate analysis of variance in the general linear model, improvement effects of the dependent variables of the pre- and post-tests of various combinations were compared.
- (3) On the bases of the F-value, p-value, and effect size ɳp2 of the repeated measures ANOVA in the general linear model, differences in the change of the dependent variable between groups of various combinations were tested.

## Results

### Subjects and demographic sociological variables

Table 1 summarizes 152 valid subjects who completed this study’s experiment, with age range of 4–6 years and average age of 4.5 years. The teacher’s questionnaire indicated that the educational level of the subjects’ parents were college and below, bachelor’s degree, and graduate degree and above, accounting for 27.0%, 57.2%, and 15.8%, respectively, of the valid subject amount (fathers); and 22.4%, 56.6%, and 21.2% (mothers), respectively. Of all selected subjects, 87.5% were rated by their teachers as doing physical activity at least thrice a week in their daily lives, 42.2% were rated by their teachers as watching nature videos or pictures at least thrice a week, and 69.1% were rated by their teachers as having a good level of attention in their daily lives.

**Table 1:** Descriptive statistics of participant age, gender, and other covariate factors

		**PA + VID**	**VID + PA**	**PA**	**VID**
**Age**	**Mean ± Standard deviation**	5.0 ± 0.52	4.5 ± 0.50	4.21 ± 0.43	4.26 ± 0.45
**Family’s financial situation**	**Poor**	0	1 (2.6%)	0	0
	**Generally**	9 (23.7%)	31 (81.6%)	33 (86.6%)	38 (100%)
	**Very good**	29 (76.3%)	6 (15.8%)	5 (13.3%)	0
**Father’s education**	**College and below**	5 (13.2%)	10 (23.6%)	14 (36.8%)	12 (31.6%)
	**Undergraduate**	20 (52.6%)	22 (57.9%)	19 (50.0%)	26 (68.4%)
	**Graduate and above**	13 (34.2%)	6 (15.8%)	5 (13.2%)	0
**Mother’s education**	**College and below**	4 (10.5%)	8 (21.1%)	12 (31.6%)	10 (26.3%)
	**Undergraduate**	20 (52.6%)	18 (47.4%)	20 (52.6%)	28 (73.7%)
	**Graduate and above**	14 (36.8%)	12 (31.6%)	6 (15.8%)	0
**Physical activity per week**	**Inactive**	0	0	0	0
	**1 time**	0	0	1 (2.6%)	0
	**2–3 times**	10 (23.6%)	5 (13.2%)	1 (2.6%)	2 (5.3%)
	**4–5 times**	13 (24.2%)	8 (21.1%)	9 (23.7%)	32 (84.2%)
	**6–7 times**	15 (39.5%)	25 (85.8%)	27 (71.1%)	4 (10.5%)
**Duration of each activity**	**Below 10 min**	2 (5.3%)	0	1 (2.6%)	0
	**15–20 min**	9 (23.7%)	2 (5.3%)	4 (10.5%)	3 (7.9%)
	**30–40 min**	24 (63.2%)	31 (81.6)	16 (42.1%)	22 (57.9%)
	**At least 1 h**	3 (7.9%)	5 (13.2%)	17 (44.7%)	13 (34.2%)
**Daily level of attention**	**Difference**	1 (2.6%)	1 (2.6%)	8 (21.1%)	0
	**Poor**	11 (28.9%)	19 (50.0%)	6 (15.8%)	1 (2.6%)
	**Good**	22 (57.9%)	11 (28.9%)	17 (44.7%)	33 (86.8%)
	**Very good**	4 (10.5%)	7 (18.4%)	7 (18.4%)	4 (10.5%)
**Number of views of natural environment videos or pictures per week**	**Do not watch**	1 (2.6%)	0	0	0
	**1 time**	0	9 (23.7%)	8 (21.1%)	0
	**2–3 times**	21 (55.3%)	21 (55.3%)	17 (44.7%)	11 (68.4%)
	**4–5 times**	15 (39.5%)	8 (21.1%)	10 (26.3%)	26 (68.4%)
	**6–7 times**	1 (2.6%)	0	3 (7.9%)	1 (2.6%)
**Duration of each viewing**	**2–5 min**	24 (63.2%)	2 (5.3%)	9 (23.7%)	9 (23.7%)
	**5–10 min**	6 (15.8%)	23 (60.5%)	14 (36.8%)	3 (7.9%)
	**10–15 min**	8 (21.1%)	13 (34.2%)	6 (15.8%)	22 (57.9%)
	**At least 20 min**	0	0	9 (23.7%)	4 (10.5%)

PA + VID:Activity + Video Group;VID + PA:Video + Activity Group;PA:Physical activity group;VID:Watch the video group

### Experimental results of the differences between the pre- and post-tests of attention in each group

#### Physical activity + natural video group

The activity + video group is scheduled to do a physical activity, take a 2-min break, and view a nature video thereafter. The difference in auditory attention is presented in Table 2. A significant difference was noted between the pre- and post-test scores of the subjects (*F* = 10.828; ɳp2 = 0.226; *p* = 0.002). The score of 9.53 ± 6.05 increased to 12.39 ± 5.40 of the post-test. For visual attention, pretest scores of the subjects in this group increased from 18.37 ± 8.49 points to 20.47 ± 6.36 points. Moreover, there was a significant difference between the pre- and post-tests (*F* = 4.287; ɳp2 = 0.104; *p* = 0.045).

**Table 2:** Test results of the pre- and post-test differences in attention within and between groups in each group

		PA + VID	VID + PA	PA	VID	Comparison between groups
Auditory attention	Pre-test	9.53 ± 6.05	10.32 ± 5.42	11.26 ± 5.91	11.55 ± 5.02	F = 1.332 *ɳp*^2^ = 0.026 *p* = 0.266
	Post test	12.39 ± 5.40	12.92 ± 5.45	11.87 ± 4.82	13.95 ± 3.82	
	Within-group comparison	F = 10.828	F = 7.213	F = 0.426	F = 9.452	
		*ɳp*^2^ = 0.226	*ɳp*^2^ = 0.163	*ɳp*^2^ = 0.011	*ɳp* ^2^ = 0.203	
		*p* = 0.002**	*p* = 0.011*	*p* = 0.518	*p* = 0.004**	
Visual attention	Pre-test	18.37 ± 8.49	19.79 ± 5.68	19.53 ± 6.80	19.95 ± 7.19	F = 2.008 ɳp^2^ = 0.051 *p* = 0.004**
	Post test	20.47 ± 6.36	16.61 ± 7.62	18.18 ± 7.56	18.42 ± 6.85	
	Within-group comparison	F = 4.287	F = 9.247	F = 0.003	F = 2.008	
		*ɳp*^2^ = 0.104	*ɳp*^2^ = 0.20	*ɳp* ^2^ = 0.051	*ɳp*^2^ = 0.051	
		*p* = 0.045*	*p* = 0.004**	*p* = 0.165	*p* = 0.165	

^*^ indicates that the effects of different physical activities on the dependent variable before and after intervention in the multivariate ANOVA model are significant, where *: *p* < 0.05; **: *p* < 0.01; ***: *p* < 0.001

PA + VID:Activity + Video Group;VID + PA:Video + Activity Group;PA:Physical activity group;VID:Watch the video group

#### Natural video + physical activity group

The activity + video group and video + activity group were simultaneous intervention activities of physical activity and exposure to green and blue spaces. This group first watched the natural environment video and performed physical activities thereafter. A significant difference was observed in the pre- and post-test scores of auditory attention (*F* = 7.213; ɳp2 = 0.163; *p* = 0.011). Pre-test scores of auditory attention increased from 10.32 ± 5.42 to 12.92 ± 5.45. Visual attention pre- and post-test scores were also significantly different (*F* = 9.247; ɳp2 = 0.20; *p* = 0.004), but the former decreased from 19.79 ± 5.68 to 16.61 ± 7.62.

#### Physical activity-based group

The 10-min intervention activities in this group were mainly physical activities. Table 2 shows no significant difference in the pre- and post-test scores of auditory attention in the physical activity group (*F* = 0.426; ɳp2 = 0.011; *p* = 0.518). However, pre-test score increased from 11.26 ± 5.91 to 11.87 ± 4.82. No significant difference was noted in the pre- and post-test scores of visual attention (*F* = 2.003; ɳp2 = 0.051; *p* = 0.165), but pre-test score decreased from 19.53 ± 6.80 to 18.18 ± 7.56.

#### Watch the video-based group

**Fig. 11:**
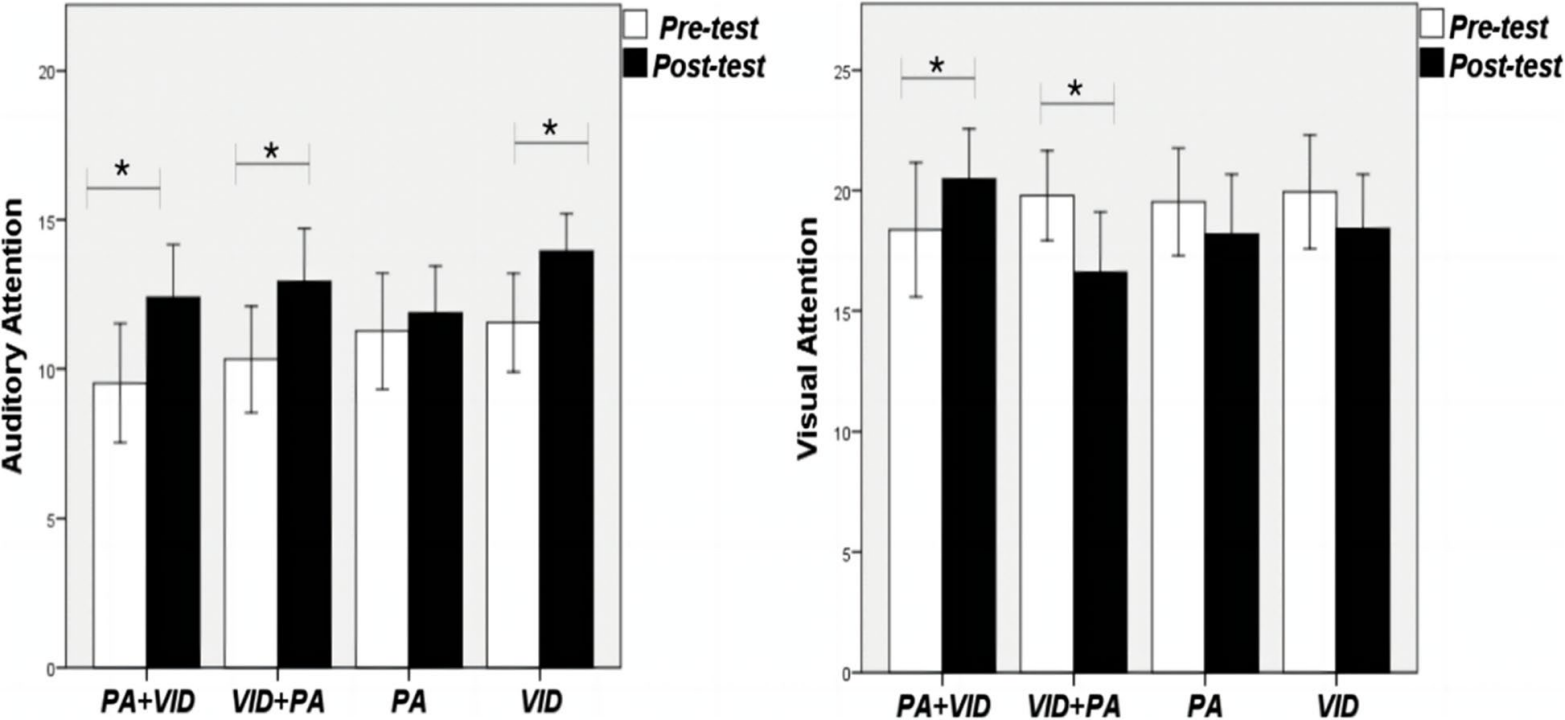
Change trend of attention pre- and post-tests in each group. * indicates that the effects of different physical activities on the dependent variable before and after intervention in the multivariate ANOVA model are significant, where *: *p* < 0.05; **: *p* < 0.01; ***: *p* < 0.001

Intervention activities in this group mainly focused on watching natural videos. Table 2 shows a significant difference in the auditory attention of the subjects in the video watching group before and after the test (*F* = 9.452; ɳp2 = 0.203; *p* = 0.004). Measured score increased from 11.55 ± 5.02 to 13.95 ± 3.82. No significant difference was observed between the pre- and post-test scores of visual attention (*F* = 2.008; ɳp2 = 0.051; *p* = 0.165). However, pre-test score decreased from 19.95 ± 7.19 to 18.42 ± 6.85. Figure 11 shows the results.

### Differences in the pre- and post-tests of attention between groups

#### Differences between auditory attention groups

Table 2 shows that in terms of auditory attention, scores of the four groups indicated an upward trend, and overall changes among the four groups did not reach a significant difference (*F* = 1.332; ɳp2 = 0.026; p = 0.266). In particular, scores of the activity + video, video + activity, and watching video groups showed significant upward changes. However, the upward trend of the physical activity group was relatively slow.

### Comparison of differences in visual attention between groups

Table 2 shows that in terms of visual attention, the comparison among the four groups of score changes reached a significant difference (*F* = 2.008; ɳp2 = 0.051; *p* = 0.004). However, only the activity + video group showed an upward trend and reached a significant difference. In addition, scores of the video + activity, physical activity, and watching video groups decreased. In particular, the decline in the video + activity group was the most significant.

### Comparison of the differences in the effect of each covariate on attention

#### Comparison of the effects of each covariate on the results between attention groups before and after adjustment

To compare the effect of each covariate on the attention level of young children, all covariates were adjusted for auditory and visual attention between groups. Table 3 shows that in terms of auditory attention, the three covariates of age, duration of each activity, and duration of each viewing have a significant impact on the results. For visual attention, the seven covariates of age (years), educational level of parents, number of physical activities per week, duration of each activity, daily attention level, number of views of natural environment videos or pictures per week, and duration of each viewing have significant effects on attention results.

**Table 3:** Comparison of the effects of each covariate on attention before and after adjustment

**Covariates**	**Auditory attention**						**Visual attention**					
	**Before fixing**			**Adjusted**			**Before fixing**			**Adjusted**		
	***F***	***ɳp***^***2***^	***P***	***F***	***ɳp***^***2***^	***P***	***F***	***ɳp***^***2***^	***P***	***F***	***ɳp***^***2***^	***P***
**Age**	1.332	0.026	0.266	0.951	0.019	0.418	2.008	0.051	**0.004**	3.949	0.075	**0.010**
**Family’s financial situation**	1.332	0.026	0.266	1.247	0.025	0.295	2.008	0.051	**0.004**	1.832	0.036	0.144
**Father’s education**	1.332	0.026	0.266	1.224	0.024	0.303	2.008	0.051	**0.004**	3.801	0.072	**0.012**
**Mother’s education**	1.332	0.026	0.266	1.234	0.025	0.299	2.008	0.051	**0.004**	4.467	0.084	**0.005**
**Physical activity per week**	1.332	0.026	0.266	1.392	0.028	0.247	2.008	0.051	**0.004**	4.558	0.085	**0.004**
**Duration of each activity**	1.332	0.026	0.266	1.713	0.034	0.167	2.008	0.051	**0.004**	4.673	0.087	**0.004**
**Daily level of attention**	1.332	0.026	0.266	1.378	0.027	0.252	2.008	0.051	**0.004**	4.665	0.087	**0.004**
**Number of views of natural environment videos or pictures per week**	1.332	0.026	0.266	1.311	0.026	0.273	2.008	0.051	**0.004**	4.240	0.080	**0.007**
**Duration of each viewing**	1.332	0.026	0.266	1.867	0.037	0.138	2.008	0.051	**0.004**	5.299	0.098	**0.002**

^*^ indicates that the effects of different physical activities on the dependent variable before and after intervention in the multivariate ANOVA model are significant, where *: *p* < 0.05; **: *p* < 0.01; ***: *p* < 0.001

### Test results of adjusted attention within each group and between groups

Table 3 shows that covariates with more significant effects were adjusted. Table 4 indicates that the adjusted data sets produce some changes. For auditory attention, greater changes were in the activity + video group (*F* = 0.022; ɳp2 = 0.001; *p* = 0.882), video + activity group (*F* = 0.214; ɳp2 = 0.006; *p* = 0.646), and viewing video group (*F* = 1.304; ɳp2 = 0.037; *p* = 0.261), none of which were significantly different. For visual attention, variation was greater in the first two groups, particularly in the video + activity group (*F* = 0.088; ɳp2 = 0.003; *p* = 0.769), and there was also no significant difference in the activity + video group (*F* = 0.264; ɳp2 = 0.009; *p* = 0.611). The greater change between groups was in visual attention (*F* = 2.402; ɳp2 = 0.049; *p* = 0.070), which was not significantly different. The experimental results indicated that after adjusting for covariates, the groups that originally had significant differences lost their significance, particularly the video + activity and activity + video groups.

**Table 4:** Difference test results of attention pre- and post-tests after adjusting for covariates within each group

		PA + VID	VID + PA	PA	VID	Comparison between groups
Auditory attention	Pre-test	9.53 ± 6.05	10.32 ± 5.42	11.26 ± 5.91	11.55 ± 5.02	F = 1.109 *ɳp*^*2*^ = 0.022 *p* = 0.348
	Post test	12.39 ± 5.40	12.92 ± 5.45	11.87 ± 4.82	13.95 ± 3.82	
	Within-group comparison	F = 0.022 *ɳp*^*2*^ = 0.001 *p* = 0.882	F = 0.214 *ɳp*^*2*^ = 0.006 *p* = 0.646	F = 3.485 *ɳp*^*2*^ = 0.093 *p* = 0.071	F = 1.304 *ɳp*^*2*^ = 0.037 *p* = 0.261	
Visual attention	Pre-test	18.37 ± 8.49	19.79 ± 5.68	19.53 ± 6.80	19.95 ± 7.19	F = 2.402 *ɳp*^*2*^ = 0.049 *p* = 0.070
	Post test	20.47 ± 6.36	16.61 ± 7.62	18.18 ± 7.56	18.42 ± 6.85	
	Within-group comparison	F = 0.264 *ɳp*^*2*^ = 0.009 *p* = 0.611	F = 0.088 *ɳp*^*2*^ = 0.003 *p* = 0.769	F = 0.365 *ɳp*^*2*^ = 0.012 *p* = 0.551	F = 0.110 *ɳp*^*2*^ = 0.004 *p* = 0.742	

On the bases of the tests of the ANOVA repeated measures, the preceding models were adjusted for age, duration of each activity, and duration of each viewing. Increased visual attention adjusted for father’s education, mother’s education, weekly physical activity, daily attention levels, and weekly viewing of videos or pictures of the natural environment

PA + VID:Activity + Video Group;VID + PA:Video + Activity Group;PA:Physical activity group;VID:Watch the video group

## Discussion

### Comparative analysis of the differences between pre- and post-tests of attention in each group

The experimental results indicated that the difference between the pre- and post-test attention scores of the auditory and visual subjects in the activity + video group was very significant, and both obtained an effective improvement. The use of VR technology to virtualize natural environmental scenes to improve attention has been confirmed by numerous studies. Rodrigo et al. (2017) observed the effects of a VR game training program on children’s reading performance, visual attention, motor balance, and coordination through a controlled experimental design. The experimental results showed a significant increase in the level of visual attention before and after training in each group (*p* = 0.042). The results showed that the training protocol of VR had a significant effect on visual attention and motor speed of participants in each group [31]. Dillon et al. (2022) conducted a study to test the impact of virtual environments with different immersion levels and scene types on attention levels. The results of ANOVA showed that participants experienced more presence in some exposures than others (*F* = 183.89, *p* < 0.001, ɳp2 = 0.61). The results also showed that the virtual natural environment scenario provides significantly superior directed attention improvement and high presence rates [13]. In recent years, there has been a growing body of research related to the effects of self-regulation through physical activity in preschool children, thereby resulting in improved cognitive performance. Nuria et al. (2020) studied 49 preschoolers aged 4–5 years with varying degrees of difficulty in 15-min recess exercise breaks, and their experiment showed a general intervention effect in all preschoolers (*F* = 11.683, *p* < 0.001, ɳp2 = 0.438) [32]. This research has focused on improving cognitive performance through a single physical activity or virtual scenario, and there is a lack of research related to different combinations of the two. On the basis of this study and accompanied by a certain degree of exploration, four different representative relaxation methods were designed to compare the best relaxation methods through controlled experiments. The experimental results of this group showed that the combination of 4 min of physical activity followed by 2 min of meditation and rest, and then 4 min of viewing the natural environment video was highly conducive to the improvement of children’s real-time sustained attention. Moreover, the virtual natural environment viewing after a short period of physical activity was clearly conducive to physical and mental relaxation and attention recovery. The combination of video viewing followed by physical activity is good for auditory attention, but worse for visual attention; the third combination with a high proportion of physical activity is also bad for attention and can even worsen it [21]. Long physical activity schedules during recess can lead to a lack of necessary quiet recovery time before class. Hence, this type of combination is not effective in improving children’s attention span.

In recent years, numerous studies have addressed the temporal continuity of visual attention in immersive VR. Moreover, an analytical discussion of the free-viewing gaze data set has suggested that temporal continuity performs well in free-viewing condition only for markedly short time intervals. Further exploration has revealed that current VR technology can be effectively applied to short-term gaze tasks, while long-term gaze tasks remain to be explored [33]. Research has suggested that the combination of VR and exercise for short periods may yield some psychological improvements compared with VR or exercise alone, and may increase some cognitive and emotional benefits, such as enhanced enjoyment, energy, reduced fatigue, and concentration [34]. This result also suggests that the combination of physical activity and VR will be markedly conducive to improved attention. Experimental results and analyses of previous studies have indicated that the fourth type of relaxation, which mainly uses VR technology to view virtual scenes of the natural environment, did not have a significant improvement effect on visual attention level. This result suggests that video viewing time for young children during relaxation should not be considerably long [35], and the specific effective viewing time remains debatable. However, the fourth type of combination, which is arranging longer natural video viewing, is helpful for the effective enhancement of auditory attention. However, further analysis and exploration are needed on the in-depth effects of physical activity and relaxation means of viewing nature videos using VR technology on young children’s auditory and visual attention, as well as the effects of changes between different combinations and different sensory attention.

### Comparison of differences in attention pre- and post-tests between groups

#### Comparative analysis of differences in auditory attention between groups

According to the experimental results, three of the four different combinations showed significant improvements in auditory attention, providing ample evidence that physical activity in fully immersive VR enhances the viewing experience and also improves cognitive abilities [15]. Campillo et al. (2016) studied the effects of brief visual and auditory interventions on performance on visual and auditory attention and memory tasks by randomly assigning 50 healthy volunteers to two brief interventions: applying visual and auditory stimuli. The results showed that auditory and visual attentional tendencies improved, with the auditory form presenting markedly immediate and effective attentional performance [36]. The results of this study found that auditory attention showed a more effective improvement than visual attention. This finding may be related to the cognitive function of the brain cortex activated by the different senses. The level of attention of different senses has been shown to be determined by the function of different cortices, with auditory tasks activating auditory, inferior parietal, prefrontal, and anterior cingulate cortices; and visual tasks activating visual association, inferior parietal, and prefrontal cortices [37]. Apparently, subjects activated different cognitive functional cortices of the brain while performing auditory and visual attention test tasks. All three sets of different combinations are effective in activating the auditory cortex and provide real-time improvement. The combination of visual sensory-based video viewing can also improve children’s auditory attention, suggesting that different sensory cortices can also play a linking role. The experimental results of this study showed that a considerably long schedule of physical activities during recess relaxation resulted in a decrease in children’s auditory attention levels.

### Comparative analysis of differences in visual attention between groups

According to the experimental results, only the combination of activity + video group had a real time improvement on children’s visual attention level among the four different combinations of relaxation activities. The video + activity group shifted the order of the two intervention activities and scheduled the physical activity in the second half, but obtained substantially different results, with a significant decrease in the subjects’ visual attention levels. Haghgoo et al. (2020) investigated the effects of a visual tracking intervention on children’s attention. They recruited 39 boys aged 6 to 10 years, randomized to receive the intervention (control group), and the intervention was accompanied by visual tracking movements (experimental group). The results of the experiment revealed a significant increase in the mean score of cognitive problems in children (*p* < 0.01) and a relative link between the motor-ocular muscle function of the visual system and cognitive function [38]. Studies have shown that the brain, while performing visual attention tasks, activates the dorsal and ventral streams of the visual pathway and posterior parietal cortex [19]. Shaea et al. (2020) found that VR without exercise increased participants’ tension and fatigue, and that using VR glasses between exercise sessions was effective in improving men’s mood and relieving stress [39]. From the experimental results of this study, the appropriate exposure to the natural environment using VR technology can significantly improve the attention level of young children, but the duration should not be considerably long, otherwise it will lead to deterioration of attention. In addition, prolonged physical activity at rest time or scheduling of physical activity items later in the day can affect children’s visual attention levels.

The results of this study are based on the attention level test of different senses, yielding completely different experimental results. Young children’s attention span and concentration change rapidly, and any small factor may interfere with the real-time attention level of the subjects. This study substantially avoided interference factors in the experimental process. The internal impact mechanism of different relaxation methods on children’s visual and auditory attention must be further explored. Changes in children’s real-time attention cannot represent the whole. Cognitive function level of children still needs continuous exploration and research on the means and methods to improve their attention.

### Comparative analysis of differences on the effects of each covariate on attention

This study used the statistical method of randomized controlled experimental grouping and repeated measures ANOVA. Theoretically, the entire experimental process can effectively avoid the differences of the subjects’ individual factors, but the influence of various covariates on the experimental results should be further investigated. Results of a randomized experimental design conducted by Ha et al. (2021) showed that a significant interaction between high biodiversity and students’ psychological recovery (attention recovery) in visual and auditory environments. Studies have also found that parents’ educational methods and family culture may affect young children’s attention levels [40, 41]. Table 3 shows seven covariates that can affect the recovery and improvement of attention. Children aged 3 to 6 years have incomplete brain development and unbalanced development of the excitation and inhibition processes of the nervous system [42], and the cognitive ability of children one year older will be significantly improved. Including the indirect effects of daily attention and physical activity levels, subjects with better attention level and physical fitness foundation will easily complete the test task than other subjects, as well as show better attention recovery ability. In addition, children who often watch nature videos will be easily adapted to the test tasks in this experiment. Lastly, other relevant factors should be further explored.

### Advantages

Searching, summarizing, and sorting out domestic and foreign studies indicated that physical activity intervention for young children can help improve attention stability, attention span, attention distribution, and attention transfer. Moreover, exposure to blue space helps improve concentration levels. Both interventions have been confirmed by numerous studies. From the perspective of different combinations of physical activity intervention and short-term viewing of natural environment videos, this study used VR technology and designed a controlled experimental study on children’s relaxation intervention to improve attention in different combinations during recess. On this basis, this research designed a controlled experimental study of different combinations of physical activity interventions and short-time video viewing of the natural environment to improve children’s attention during recess. The use of VR technology for limited exposure to green and blue space enriches the comparison of different combinations of improvement effects that have not been adequately conducted in the current research in this area, helping children to improve their attention easily, immediately, and effectively. Therefore, the research design of the different combinations of physical activity and viewing natural environment videos to improve children’s attention effects and the use of VR technology to view the natural environment are the characteristics and innovations of this research.

### Limitations

- (1) Given that the continuous test method for young children in this study is real-time and the attention of young children is in the budding stage, attention remains scattered and any subtle interference factors during the experiment may affect the subjects’ real-time attention. The test results will continue to deepen and refine the relevant aspects of this research in the follow-up to constantly improve the test methods, optimize the experimental site and equipment, and minimize interference factors in the test process, thereby ensuring the authenticity of the experimental results.
- (2) Subjects recruited in this study are young children from cities, and the kindergartens they choose are college-affiliated kindergartens. Their family living environment, economic conditions, and educational methods are relatively good, and their cognitive function and learning ability will also be affected by cities. The influence of cultural and living environment will be better than other preschool children. However, the COVID-19 pandemic had prevented the recruitment of subjects from a markedly wide social group, thereby resulting in the experiment’s certain limitations. In future research, there is optimism that academic ability will be continuously improved and further research will continue in related aspects, such as green fitness for young children.
- (3) This study’s experimental intervention adopts high-tech VR technology, which has yet to mature mainly owing to the instability of network data, poor image, and lack of overall participation in the natural environment. Compared with the real natural environment, a huge gap is noted, thereby affecting the viewing experience of children to varying degrees. Evidently, the virtual artificial scene cannot be compared with the real nature. However, this study firmly believes that with the continuous development of human technology in the near future, the former can approximate real nature, thereby ultimately benefitting mankind.

## Conclusions

Among the several interventions that can use physical activity and watching the natural environment to promote the recovery of attention levels during children’s recess, the effect of improving their attention level is the best after physical activity intervention and watching natural environment videos. Moreover, this study found that arranging physical activity interventions in the second half of children’s recess will affect their attention, particularly the visual attention level, at the beginning of class. Therefore, this study recommends not to arrange children’s physical activity intervention in the second half of the class break. Moreover, we recommend to arrange the content of physical activity intervention first and further improve their attention further by watching natural environment videos.

Among interventions using physical activity and natural environment viewing to promote recovery of attention levels, important individual factors are age, weekly physical activity frequency, duration of each physical activity, weekly natural environment video or picture viewing frequency, duration of each nature video viewing, mother’s education level, and daily attention level. Future research in this field should focus on the influence of individual factors on young children’s attention and strengthen the adjustment of related covariates.

## Data Availability

Data sets used and/or analyzed in the current study are available from the corresponding author upon reasonable request.

## Abbreviations

ANOVA: Repeated measures of analysis of variance
VR: Virtual reality
